# Editorial Bias in Crowd-Sourced Political Information

**DOI:** 10.1371/journal.pone.0136327

**Published:** 2015-09-02

**Authors:** Joshua L. Kalla, Peter M. Aronow

**Affiliations:** 1 Charles and Louise Travers Department of Political Science, University of California, Berkeley, Berkeley, California, United States of America; 2 Departments of Political Science and Biostatistics, Yale University, New Haven, Connecticut, United States of America; University of Warwick, UNITED KINGDOM

## Abstract

The Internet has dramatically expanded citizens’ access to and ability to engage with political information. On many websites, any user can contribute and edit “crowd-sourced” information about important political figures. One of the most prominent examples of crowd-sourced information on the Internet is Wikipedia, a free and open encyclopedia created and edited entirely by users, and one of the world’s most accessed websites. While previous studies of crowd-sourced information platforms have found them to be accurate, few have considered biases in what kinds of information are included. We report the results of four randomized field experiments that sought to explore what biases exist in the political articles of this collaborative website. By randomly assigning factually true but either positive or negative and cited or uncited information to the Wikipedia pages of U.S. senators, we uncover substantial evidence of an editorial bias toward positivity on Wikipedia: Negative facts are 36% more likely to be removed by Wikipedia editors than positive facts within 12 hours and 29% more likely within 3 days. Although citations substantially increase an edit’s survival time, the editorial bias toward positivity is not eliminated by inclusion of a citation. We replicate this study on the Wikipedia pages of deceased as well as recently retired but living senators and find no evidence of an editorial bias in either. Our results demonstrate that crowd-sourced information is subject to an editorial bias that favors the politically active.

## Introduction

In a democracy, a well-informed electorate is necessary to hold politicians accountable [1]. This is facilitated in part by information provided by the media [2–6]. Yet traditional media sources, such as newspapers and broadcast news, have been found to display ideological and partisan biases in the information they present [7–13]. Critics of these traditional media sources have hoped that the increase in political information available on the Internet, and in particular via democratized, crowd-sourced platforms, would provide a powerful medium for questioning political authority [14,15].

Internet-based crowdsourcing enables millions of individuals to contribute small amounts of effort to a much larger enterprise [16,17]. One of the most prominent examples of crowdsourcing is Wikipedia–an open and free encyclopedia where any user can contribute and edit articles. Launched in 2001, Wikipedia today has over 4.6 million English-language articles and continues to grow at a rate of over 800 new articles per day [18]. Wikipedia is currently the sixth most visited website in the United States [19] and has become a common source of information about politics, as well as more general topics. For searches about politicians on Google, Wikipedia is frequently the second website listed, behind a politician’s official website. Importantly, when voters turn to Wikipedia as a source, they perceive it as trustworthy [20].

In this paper, we examine what kinds of biases exist in crowd-sourced information on Wikipedia. Because any user can also be an editor, no one person with any particular ideological or partisan motivation should be able to control Wikipedia [21]. Yet scholars have recognized the tension that comes from distributed, crowd-sourced platforms like Wikipedia. On the one hand, crowd-sourced information platforms create opportunities for citizens to challenge media-driven narratives. On the other, these platforms may be captured by the self-interested who have the greatest motivation to shape them for their own benefit [22].

Most studies of Wikipedia have focused on the accuracy of information presented rather than on biases in what information is included in the first place. Wikipedia has been found to be as accurate as the *Encyclopedia Britannica* in its coverage of scientific subjects [23] and to correctly report specific, verifiable facts about political candidates’ biographies and election results [24]. While the information that is presented may be accurate, the types of information made available on Wikipedia may not reflect the full range of information that is available about a particular politician due to filtering and censorship [15]. For example, a Wikipedia article that correctly identifies when a politician was born and how she fared in each of her elections may be accurate–all of the statements presented are true–without providing a complete and fully useful portrait of that politician. If the article failed to mention major legislation authored by or scandals involving the politician, we might worry about biases in what kinds of information are omitted. In short, Wikipedia has fared well in evaluations of its accuracy, while at the same time it has been acknowledged to suffer from errors of omission [24–26].

We present a series of randomized field experiments in which we explore one possible mechanism leading to the lack of comprehensiveness on Wikipedia: editorial bias. Editorial bias refers to the idea that certain types of information are more likely to be removed from Wikipedia, resulting in a potentially incomplete article. Across four experiments and consistent with extant research from a social news aggregation website [27], we find strong evidence of an editorial bias toward positivity, in which negative information about active politicians is far more likely to be removed from Wikipedia than positive information.

## Methods

The complete details of the experimental methodology are described in S1–S4 Files, but are summarized briefly here. Both the Yale University Human Subjects Committee and the University of California, Berkeley Committee for Protection of Human Subjects approved the design, and the design was pre-registered with Experiments in Governance and Politics (EGAP). IRB approval by both Yale University and the University of California, Berkeley was granted under Yale University's Special Exemption Category 7: Research Involving Response to Non-Physically Invasive Stimuli. Both IRBs approved an exemption from obtaining consent because the research involved non-physically invasive stimuli with interaction entirely online that posed no more than minimal risk to participants. The authors believe that all actions taken as part of the experiments were in compliance with Wikimedia’s Terms of Use [28].

During the summer and fall of 2014, we conducted four studies on Wikipedia, all of which followed the same basic design. Studies 1, 2, and 4 were conducted on the English-language Wikipedia pages of the 100 current U.S. senators, while Study 3 was conducted on the 151 English-language Wikipedia pages of senators who have passed away or otherwise left the Senate since 1990 and do not currently hold any elective office or serve as a Cabinet Secretary.

In each study, research assistants collected one true positive and one true negative fact from a reputable news source on each senator that was not currently mentioned on that senator’s Wikipedia page. External coders were used to validate the coding of the facts as positive or negative (further details presented below and in S12 File). For each study, we then randomly assigned half of the pages to have a positive fact added to the page and the other half to have a negative fact added to the page.

In Studies 1 and 4, we used a factorial design, where we also randomized half of the pages to include a citation with the fact, and the other half without a citation. In Studies 2 and 3, we always included a citation. S1–S4 Files present randomization checks and report the expected covariate balance across the treatment conditions for each study. In S5 File, we report one concluding study to replicate Studies 1 and 4 that was partially aborted due to problems in the implementation, the inclusion of which does not change the results.

### Details on the Facts

A complete list of the facts used in these studies is available in the replication data. Research assistants were undergraduate college students who were told to find a non-policy related positive and negative fact for each senator from a reputable news source. The research assistants were blind to the hypotheses being tested and we reviewed all facts before the experiment was started. An example positive fact is “During the government shutdown in 2013 Heitkamp donated about $8,000 of her salary to North Dakota charities that support veterans, provide healthcare supplies to those that cannot afford them, and raise Breast Cancer awareness.” [29] An example of a negative fact is “The Annenberg Public Policy Center has accused Murray of lying to voters by insisting that if they liked their health insurance plan, they would be able to keep it” [30].

After we completed all four studies, we used Amazon Mechanical Turk to evaluate how the facts were perceived [31]. Five Mechanical Turk survey respondents rated each fact on how positive or negative it is, how relevant it is, and how favorable they feel towards the senator after reading the fact. Consistent with our expectations, we find that the Mechanical Turk survey respondents perceive the positive facts collected by the undergraduate research assistants as more positive and as leading to more favorable feelings towards the senator than the negative facts. Importantly, we find that both the positive and negative facts were perceived as equally relevant on a 7-point scale from very irrelevant to very relevant, positive facts were on average rated 5.0 (SE = 0.04) and negative facts were on average rated 5.0 (SE = 0.04). More details are presented in S12 File.

### Implementation on Wikipedia

After the facts were collected and senators were randomly assigned to experimental conditions, we inserted the facts into the corresponding senator’s Wikipedia article in the section that seemed most appropriate. In order to prevent confounding, the order in which the edits were made, the time of day, the name of the editing Wikipedia account, and the IP address used to make the edit were randomly assigned as well. With the exception of Study 1, new accounts were created before each study began and were seeded with true, cited, political, non-Senate edits to Wikipedia. More details are presented in S1–S4 Files.

### Estimation of Experimental Effects

Our primary dependent variable is the survival time of the facts–the amount of time between when the fact is added to Wikipedia and when it is removed. To assess the statistical difference in the survival times between the experimental conditions, we use a Cox proportional hazard model [32].

## Results

### Survival of Positive and Negative Facts on Wikipedia

**Fig 1:**
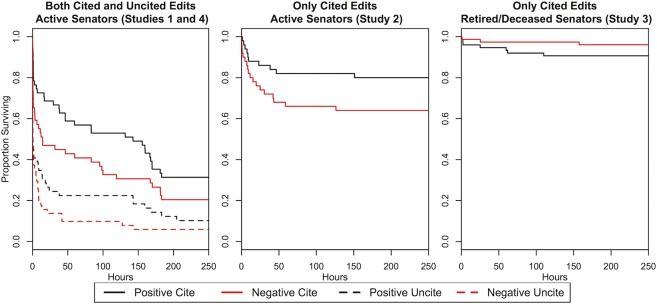
Kaplan-Meier survival curves across all studies and all conditions. Studies 1, 2, and 4 were conducted on the current U.S. senators while Study 3 was conducted using the Wikipedia pages of retired and deceased senators. Results show that among active senators, negative facts are fare more likely to be removed more quickly than positive facts.

The objective of our primary analysis is to determine whether there is a difference in how long a fact remains on the Wikipedia page of a U.S. senator depending on the valence of the fact. Consistently across Studies 1, 2, and 4, we find strong evidence of an editorial bias toward positivity on Wikipedia. Pooling across studies, negative facts are far more likely to be removed than positive facts by other Wikipedia editors (hazard ratio = 1.59, a measure of the rate at which an event happens in one treatment group over another, *p* = 0.001). (Results disaggregated by study are presented in Tables D and E and Figure A in S6 File; further robustness checks are presented in Tables A and B in S11 File) These results, as seen in Fig 1, demonstrate clear, systematic bias in what type of information remains on Wikipedia and what is removed. We find no systematic evidence that this effect is moderated by covariates (state population, Senate class, character count of page, geographic region, length of incumbency, political party, date of entry, and time of entry; see Table A in S9 File and S10 File), as specified in our registered pre-analysis plan.

### Effects of Citations on Survival

Wikipedia maintains a policy that all information must be verifiable and that edits must include citations [33]. To test this policy and whether it is selectively applied depending on the valence of the information, in Studies 1 and 4 we randomly assigned positive and negative facts to either include a citation or to have no citation (more details are provided in the SI). Overall, we find large and statistically significant differences in the survival time of cited and uncited facts. Consistent with Wikipedia’s policy, uncited (but true) facts are 64% more likely to be removed than cited facts within four hours (hazard ratio = 2.33, *p*<0.001). We find evidence that membership in a Senate class that is about to face reelection moderates the effect of citations. The difference in the survival rates between cited and uncited facts is greater for senators who faced reelection in 2014 than for senators not up for reelection that year (interaction term hazard ratio = 0.32, p<0.001, see S8 File). This suggests that Wikipedia’s citation policy is most likely to be applied when the stakes are greater. We find no systematic evidence that any other variables moderate the effect of citations, nor do we find any evidence of an interaction effect between citations and whether or not the fact was positive or negative (see Table B in S9 File and Tables A and B in S7 File).

In Study 2, all edits were cited and only differed in whether they were positive or negative. In this study, we continue to find that negative facts are removed more quickly than positive ones, but that all edits last longer on average than in the other studies. This could be because the Wikipedia accounts used to make the edits were perceived to be of higher quality since they were only used to make cited edits.

### Replication on Wikipedia Pages of Dead and Retired Senators

Wikipedia has special guidelines for articles that are biographies of living persons that require a more conservative approach to avoid libel [34]. To ensure that this more conservative approach does not explain our finding of editorial bias, Study 3 conducted a replication on the Wikipedia pages of senators who have left office since 1990. Both among senators who have left office and are still living and those who have passed away, we find no differences in the rates in which positive and negative facts are removed (*p* = 0.70 and 0.47, respectively and *p* = 0.42, pooled). If anything, the positive facts were removed more quickly than the negative ones. This suggests that the editorial bias cannot be explained by Wikipedia’s culture in general or by policies concerning biographies of living persons in particular. Instead, our findings suggest the editorial bias is limited to active politicians, the types of people for whom the stakes of a positive public image are the highest.

## Discussion

This experiment demonstrated that a substantial editorial bias exists for the Wikipedia pages of current U.S. senators. Across four studies, we consistently find that negative facts are more likely to be removed and are removed more quickly than positive facts. In a test conducted on the Wikipedia pages of recently retired and deceased senators, we find no editorial bias, suggesting that this bias is limited to the pages of active politicians and is not systemic to Wikipedia.

The reasons for the editorial bias toward positivity are not necessarily nefarious. It is plausible that Wikipedia editors are simply more conservative in applying their guidelines for living persons to active U.S. Senators than recently retired U.S. senators, even if the editing policies are nominally identical for both types of articles. The bias may also be an unconscious corrective to the amount of negative “trolling” on the Internet [35], even though the facts added to the pages were cited. As the crowd-sourced Internet continues to grow, understanding the causes behind this editorial bias and whether it exists in more settings will become an increasingly important research agenda so that the administrators of crowd-sourced websites may be able to adopt policies capable of overcoming this editorial bias.

But regardless of the causes of the editorial bias, it is important to recognize that this bias exists and that it led to true, relevant, and cited information being selectively removed from Wikipedia pages in the days and months leading up to the 2014 midterm elections. While any one edit is unlikely to have a substantial effect on public perceptions of a politician, we observed this editorial bias toward positivity across several months and four distinct studies. Biases such as this may build over time and lead to significant distortions in what information is presented [27].

## Supporting Information

S1 File (DOCX)

S2 File (DOCX)

S3 File (DOCX)

S4 File (DOCX)

S5 File (DOCX)

S6 File (DOCX)

S7 File (DOCX)

S8 File (DOCX)

S9 File (DOCX)

S10 File (DOCX)

S11 File (DOCX)

S12 File (DOCX)
