# Supplementary material for: Editorial Bias in Crowd-Sourced Political Information
Source: PLoS One. 2015 Sep 2;10(9):e0136327. doi: 10.1371/journal.pone.0136327 (PMC4558055; doi:10.1371/journal.pone.0136327)
Supplement: S1 File — (DOCX) [file pone.0136327.s001.docx]

**S1 File. Study 1.**

*Subjects*: This study was conducted using the Wikipedia pages of the 100 current U.S. senators.

*Details of Random Assignment*: Senators were first randomly assigned into 25 blocks of 4. Within each block, 2 senators were randomly assigned to receive a negative fact and 2 were randomly assigned to receive a positive fact. Independent of the type of fact, 2 senators were randomly assigned to have the fact cited and 2 senators were randomly assigned to have the fact uncited. The date, time, and account used to make the edits were randomly assigned as well . This experimental design was registered online with the online experiment registry of EGAP – Experiments in Governance and Politics.

*Table A. Covariate balance for Study 1*

|  | Positive Cited | Positive Uncited | Negative Cited | Negative Uncited |
| --- | --- | --- | --- | --- |
| Proportion Democrat | 0.5  (0.1) | 0.58  (0.1) | 0.46  (0.1) | 0.62  (0.1) |
| Senate Class | 1.77  (0.14) | 2.17  (0.18) | 2.33  (0.18) | 1.81  (0.15) |
| Years in Senate | 9.65  (1.66) | 10.46  (2.2) | 9.67  (2.18) | 11.12  (1.81) |
| State Population | 5238178  (1161001) | 7259108  (1789737) | 6203403  (1075024) | 6038259  (1393552) |
| Wikipedia Page Character Count | 224746  (15387) | 239601  (25926) | 229349  (13721) | 209554  (15895) |
| N | 26 | 24 | 24 | 26 |

*Note:* Cells report the mean followed by the standard error of the mean in parentheses. A multinomial logistic regression to predict treatment assignment as a function of the covariates confirms balance: a likelihood ratio test with 15 degrees of freedom finds χ^2^ = 14.3, *p* = 0.50.

*Description of implementation*

From 7-15 July 2014, we inserted randomly assigned facts into the Wikipedia pages of U.S. senators. The time, order, Wikipedia account, valence (positive or negative), and citation (or not) were all randomly assigned. Edits were inserted into the section of the article that seemed most appropriate for the fact. If there was no appropriate subsection, one was created. If the fact was to be cited, standard Wikipedia citation protocols were followed. After all edits were made, we tracked how long it took for the fact to be removed.

While implementing the experiment, we did not anticipate that the pages of certain U.S. senators would be locked. The pages of Rand Paul, Elizabeth Warren, Jim Inhofe, Tammy Baldwin, Bob Menendez, Dianne Feinstein, Marco Rubio, Ted Cruz, Mark Kirk, and John McCain are currently to be “semi-protected,” meaning they cannot be edited by users who have not been autoconfirmed. To become an autoconfirmed user, an account must be at least four days old and must have made at least ten edits.

We encountered our first semi-protected page, Rand Paul’s, after making our first two edits. We continued to make the remaining edits for day one, but then encountered two more semi-protected pages. After examining the number of semi-protected pages, we decided to modify the experimental procedure. All subsequent edits were made from the same account, veto118. This account was randomly assigned to be the first account. We then made ten non-political edits using this account, allowing us to move closer to achieving autoconfirmed status. When we came across a semi-protected page, we skipped this page until we achieved autoconfirmed status. Once we received that status, we went back and made the edits to the earlier skipped semi-protected pages. We also continued to make one non-political edit per day alongside the edits included in this experiment. As soon as we encountered the problem of the locked pages, we decided on this change in the experimental protocol and submitted an addendum to EGAP.

Because other Wikipedia editors became suspicious of this account’s uncited edits and threatened to block the account, we sped up the implementation and finished implementing the experiment sooner than anticipated, on 15 July 2014. Because the order in which edits were made was randomly assigned, this does not bias our results.
