# Supplementary material for: Editorial Bias in Crowd-Sourced Political Information
Source: PLoS One. 2015 Sep 2;10(9):e0136327. doi: 10.1371/journal.pone.0136327 (PMC4558055; doi:10.1371/journal.pone.0136327)
Supplement: S2 File — (DOCX) [file pone.0136327.s002.docx]

**S2 File. Study 2.**

*Subjects*: This study was conducted using the Wikipedia pages of the 100 current U.S. senators.

*Details of Random Assignment*: Senators who received a positive fact in Study 1 received a negative fact in Study 2 and vice-versa. The uncited condition was dropped and all facts were cited. The date, time, IP address (using two university VPNs and current location, only introduced beginning in Study 2) and account used to make the edits were randomly assigned as well. All of these changes were noted as an addendum to our original EGAP registration.

*Table A. Covariate balance for Study 2*

|  | Positive Cited | Negative Cited |
| --- | --- | --- |
| Proportion Democrat | 0.54  (0.07) | 0.54  (0.07) |
| Senate Class | 2.06  (0.12) | 1.96  (0.11) |
| Years in Senate | 10.42  (1.4) | 10.04  (1.35) |
| State Population | 6117528  (880762) | 6208224  (1048860) |
| Wikipedia Page Character Count | 219056  (10557) | 231876  (14676) |
| N | 50 | 50 |

*Note:* Cells report the mean followed by the standard error of the mean in parentheses. A multinomial logistic regression to predict treatment assignment as a function of the covariates confirms balance: a likelihood ratio test with 5 degrees of freedom finds χ^2^ = 1.16, *p* = 0.95.

*Description of implementation*

From 13-16 August 2014, we inserted randomly assigned facts into the Wikipedia pages of U.S. senators. The time, order, Wikipedia account, IP address, and valence (positive or negative) were all randomly assigned. Edits were inserted into the section of the article that seemed most appropriate for the fact. If there was no appropriate subsection, one was created. After all edits were made, we tracked how long it took for the fact to be removed.

After learning about the semi-protected locked pages in Study 1, we decided to be proactive in order to achieve autoconfirmed status. Five days before starting the experiment, we created five Wikipedia accounts and made two edits per day with each account on topics related to American politics but unrelated to senators or their elections. This allowed us to reach autoconfirmed status on all accounts, granting us the ability to edit locked pages. We then made 25 edits per day from the five accounts, with the time, order, account, IP address, and valence of fact all randomly assigned. No implementation problems were encountered.
