# Supplementary material for: Editorial Bias in Crowd-Sourced Political Information
Source: PLoS One. 2015 Sep 2;10(9):e0136327. doi: 10.1371/journal.pone.0136327 (PMC4558055; doi:10.1371/journal.pone.0136327)
Supplement: S3 File — (DOCX) [file pone.0136327.s003.docx]

**S3 File. Study 3**

*Subjects*: This study was conducted using the Wikipedia pages of senators who have passed away since 1990 or are living and retired during or before 2009. 243 senators fit these criteria, but research assistants were only able to collect positive and negative facts for 151 of these senators.

*Details of Random Assignment*: These Wikipedia pages were randomly assigned to receive either a positive or negative fact (all cited). The date, time, IP address, and account used to make the edits were randomly assigned as well. All of these changes were noted as an addendum to our original EGAP registration.

*Table A. Covariate balance for Study 3*

|  | Positive Cited | Negative Cited |
| --- | --- | --- |
| Proportion Democrat | 0.47  (0.06) | 0.49  (0.06) |
| Still Alive | 0.45  (0.06) | 0.50  (0.06) |
| Last Year in Senate | 1988.7  (1.69) | 1988.9  (1.49) |
| N | 75 | 76 |

*Note:* Cells report the mean followed by the standard error of the mean in parentheses. A multinomial logistic regression to predict treatment assignment as a function of the covariates confirms balance: a likelihood ratio test with 3 degrees of freedom finds χ^2^ = 0.46, *p* = 0.93.

*Description of implementation*

From 3-14 October 2014, we inserted randomly assigned facts into the Wikipedia pages of dead and retired U.S. senators. The time, order, Wikipedia account, IP address, and valence (positive or negative) were all randomly assigned. Edits were inserted into the section of the article that seemed most appropriate for the fact. If there was no appropriate subsection, one was created. After all edits were made, we tracked how long it took for the fact to be removed.

After learning about the semi-protected locked pages in Study 1, we decided to be proactive in order to achieve autoconfirmed status. We created 5 Wikipedia pages and made around 20 political, non-Senate edits using these accounts before beginning the experiment. No implementation problems were encountered.
