# Supplementary material for: Editorial Bias in Crowd-Sourced Political Information
Source: PLoS One. 2015 Sep 2;10(9):e0136327. doi: 10.1371/journal.pone.0136327 (PMC4558055; doi:10.1371/journal.pone.0136327)
Supplement: S5 File — (DOCX) [file pone.0136327.s005.docx]

**S5 File. Study 5. Partially Aborted Concluding Study.**

*Subjects*: This study was conducted using the Wikipedia pages of the 100 current U.S. senators.

*Details of Random Assignment*: Senators who received a positive fact in Study 4 received a negative fact here and vice-versa. The citation (cited or not) was randomly assigned independent of valence. The date, time, IP address, and account used to make the edits were randomly assigned as well. All of these changes were noted as an addendum to our original EGAP registration.

*Table A. Covariate balance for Study 5*

|  | Positive Cited | Positive Uncited | Negative Cited | Negative Uncited |
| --- | --- | --- | --- | --- |
| Proportion Democrat | 0.54  (0.08) | 0.64  (0.15) | 0.49  (0.08) | 0.64  (0.15) |
| Senate Class | 1.90  (0.13) | 2.09  (0.28) | 2.03  (0.13) | 2.27  (0.24) |
| Years in Senate | 9.85  (1.48) | 9.45  (2.37) | 10.67  (1.76) | 10.82  (2.67) |
| State Population | 4831639  (824949) | 9357564  (2992623) | 7584134  (1212683) | 2649028  (494777) |
| Wikipedia Page Character Count | 221885  (15379) | 241232  (21068) | 235504  (13167) | 186806  (33704) |
| N | 39 | 11 | 39 | 11 |

*Note:* Cells report the mean followed by the standard error of the mean in parentheses. A multinomial logistic regression to predict treatment assignment as a function of the covariates confirms balance: a likelihood ratio test with 15 degrees of freedom finds χ^2^ = 17.91, *p* = 0.27.

*Description of implementation*

From 6-7 November 2014, we inserted randomly assigned facts into the Wikipedia pages of U.S. senators. The time, order, Wikipedia account, IP address, valence (positive or negative), and citation (cited or not) were all randomly assigned. Edits were inserted into the section of the article that seemed most appropriate for the fact. If there was no appropriate subsection, one was created. After all edits were made, we tracked how long it took for the fact to be removed.

The 90 senators without locked pages were randomly assigned to receive edits from one of 45 accounts. Each account made no more than 4 edits in total over Studies 4 and 5. The 10 senators with locked pages were randomly assigned to receive edits from one of 10 accounts. Each account made no more than 4 edits in total over Studies 4 and 5. These accounts have already been used to make edits in previous rounds of this experiment and thus were autoconfirmed. There was a surplus of edits from these locked accounts, since the 10 accounts can make 4 edits each (40), yet we only needed 20 edits from them for Studies 4 and 5. This surplus was intended in case a senator’s Wikipedia page became locked and we needed an autoconfirmed account to make edits. After implementing the first 50 edits on 6 November 2014, other Wikipedia editors became suspicious. We decided to convert all of the uncited edits in the remaining 50 edits to cited edits. Thus the first 50 edits had all four conditions while the last 50 edits only had positive or negative conditions, with all facts cited. Because the order in which edits were made was randomly assigned, this does not bias our results.
