# Supplementary material for: Editorial Bias in Crowd-Sourced Political Information
Source: PLoS One. 2015 Sep 2;10(9):e0136327. doi: 10.1371/journal.pone.0136327 (PMC4558055; doi:10.1371/journal.pone.0136327)
Supplement: S6 File — (DOCX) [file pone.0136327.s006.docx]

**S6 File. Additional Analyses and Robustness Checks.**

The Kaplan-Meier plots and regression models for all five studies are presented below in Figure A and Tables D and E. With the exception of Study 3 (presented in Table C), which included the dead and retired senators, across all studies we find consistent evidence of an editorial bias favoring positive facts and cited facts. Pooled results showing that positive and cited facts are more likely to survive are presented in Tables A and B.

*Figure A. Kaplan-Meier plots from all five studies.*

**Table A: Pooled Cox Regression Estimates**

**Coefficient:**

**Positive**

*Coefficient:* 0.629***

*Standard error:* (0.091)

**Cited**

*Coefficient:* 0.429***

*Standard error:* (0.069)

**N**  300 200

**Fixed Effects** Yes Yes

**Covariates** No No

**Studies**  1, 2,4 1,4

Note: *Significant at the 10% level; **Significant at the 5% level; ***Significant at the 1% level; Fixed Effects refer to fixed effects for study wave; Covariates Yes means controlling for the date and time order in which an edit was randomly assigned to be made, a binary variable for Republicans, Senate class, region (NE, S, W), length of incumbency, log of Wikipedia page character count before Study 1 began, log of state population, and a dichotomous influence variable for party leaders and committee chairs.

**Table B: Covariate Adjusted Cox Regression Pooled Estimates**

**Coefficient:**

**Positive**

*Coefficient:* 0.670**

*Standard error:* (0.095)

**Cited**

*Coefficient:* 0.352***

*Standard error:*  (0.055)

**N**  300 200

**Fixed Effects** Yes Yes

**Covariates** Yes Yes

**Studies** 1,2,4 1,4

Note: *Significant at the 10% level; **Significant at the 5% level; ***Significant at the 1% level; Fixed Effects refer to fixed effects for study wave; Covariates Yes means controlling for the date and time order in which an edit was randomly assigned to be made, a binary variable for Republicans, Senate class, region (NE, S, W), length of incumbency, log of Wikipedia page character count before Study 1 began, log of state population, and a dichotomous influence variable for party leaders and committee chairs.

**Table C: Study 3 Cox Regression Estimates for Dead and Retired Senators**

**Coefficient:**

**Positive**

*Coefficient:* 0.691 0.775 0.627

*Standard error:* (0.318) (0.503) (0.406)

**N**  151 72 79

**Fixed Effects** No No No

**Covariates** No No No

**Studies**  3 3 (Retired) 3 (Dead)

Note: *Significant at the 10% level; **Significant at the 5% level; ***Significant at the 1% level; Fixed Effects refer to fixed effects for study wave; Covariates Yes means controlling for the date and time order in which an edit was randomly assigned to be made, a binary variable for Republicans, Senate class, region (NE, S, W), length of incumbency, log of Wikipedia page character count before Study 1 began, log of state population, and a dichotomous influence variable for party leaders and committee chairs.

**Table D: Study by Study Cox Regression Estimates, without Covariate Adjustment**

**Coefficient:**

**Positive**

*Coefficient:* 0.532*** 0.530* 0.857 0.580* 0.338***

*Standard error:* (0.117) (0.192) (0.213) (0.181) (0.127)

**Cited**

*Coefficient:* 0.520*** 0.358*** 0.542*

*Standard error:* (0.108) (0.087) (0.182)

**N**  100 100 100 50 50

**Fixed Effects** No No No No No

**Covariates** No No No No No

**Studies**  1 2 4 5 (1^st^ Half) 5(2^nd^ Half)

Note: *Significant at the 10% level; **Significant at the 5% level; ***Significant at the 1% level; ****Date of entry and hour of entry removed to avoid convergence issues; Fixed Effects refer to fixed effects for study wave; Covariates Yes means controlling for the date and time order in which an edit was randomly assigned to be made, a binary variable for Republicans, Senate class, region (NE, S, W), length of incumbency, log of Wikipedia page character count before Study 1 began, log of state population, and a dichotomous influence variable for party leaders and committee chairs.

**Table E: Study by Study Cox Regression Estimates, with Covariate Adjustment**

**Coefficient:**

**Positive**

*Coefficient:* 0.532*** 0.574 0.753 0.387*** 0.274*

*Standard error:* (0.108) (0.220) (0.200) (0.139) (0.162)

**Cited**

*Coefficient:* 0.455*** 0.239*** 0.480*

*Standard error:* (0.090) (0.068) (0.174)

**N**  100 100 100 50 50

**Fixed Effects** No No No No No

**Covariates** Yes Yes Yes Yes^ Yes

**Studies**  1 2 4 5 (1^st^ Half) 5 (2^nd^ Half)

Note: *Significant at the 10% level; **Significant at the 5% level; ***Significant at the 1% level; ^Date of entry, and hour of entry removed to avoid convergence issues; Fixed Effects refer to fixed effects for study wave; Covariates Yes means controlling for the date and time order in which an edit was randomly assigned to be made, a binary variable for Republicans, Senate class, region (NE, S, W), length of incumbency, log of Wikipedia page character count before Study 1 began, log of state population, and a dichotomous influence variable for party leaders and committee chairs.
