# Supplementary material for: Editorial Bias in Crowd-Sourced Political Information
Source: PLoS One. 2015 Sep 2;10(9):e0136327. doi: 10.1371/journal.pone.0136327 (PMC4558055; doi:10.1371/journal.pone.0136327)
Supplement: S7 File — (DOCX) [file pone.0136327.s007.docx]

**S7 File.** **No evidence of an interaction effect between positive and cited facts on survival time.**

**Table A: Interaction between Positive and Cited from Cox Regressions**

**Coefficient:**

**Positive**

*Coefficient:* 0.701 0.673*

*Standard error:* (0.167) (0.146)

**Cited**

*Coefficient:* 0.460*** 0.490***

*Standard error:* (0.097) (0.091)

**Positive X Cited**

*Coefficient:* 0.866 0.853

*Standard error:* (0.272) (0.242)

**N**  200 250

**Fixed Effects** Yes Yes

**Covariates** No No

**Studies** 1, 4 1,4,5 (1^st^ Half)

Note: *Significant at the 10% level; **Significant at the 5% level; ***Significant at the 1% level; Fixed Effects refer to fixed effects for study wave; Covariates Yes means controlling for the date and time order in which an edit was randomly assigned to be made, a binary variable for Republicans, Senate class, region (NE, S, W), length of incumbency, log of Wikipedia page character count before Study 1 began, log of state population, and a dichotomous influence variable for party leaders and committee chairs.

**Table B: Interaction between Positive and Cited from Cox Regressions, with Covariate Adjustment**

**Coefficient:**

**Positive**

*Coefficient:* 0.737 0.708*

*Standard error:* (0.167) (0.146)

**Cited**

*Coefficient:* 0.386*** 0.448***

*Standard error:*  (0.078) (0.079)

**Positive X Cited**

*Coefficient:* 0.818 0.779

*Standard error:*  (0.246) (0.212)

**N**  200 250

**Fixed Effects** Yes Yes

**Covariates** Yes Yes

**Studies** 1, 4 1,4,5 (1^st^ Half)

Note: *Significant at the 10% level; **Significant at the 5% level; ***Significant at the 1% level; Fixed Effects refer to fixed effects for study wave; Covariates Yes means controlling for the date and time order in which an edit was randomly assigned to be made, a binary variable for Republicans, Senate class, region (NE, S, W), length of incumbency, log of Wikipedia page character count before Study 1 began, log of state population, and a dichotomous influence variable for party leaders and committee chairs.
