# Supplementary material for: Editorial Bias in Crowd-Sourced Political Information
Source: PLoS One. 2015 Sep 2;10(9):e0136327. doi: 10.1371/journal.pone.0136327 (PMC4558055; doi:10.1371/journal.pone.0136327)
Supplement: S8 File — (DOCX) [file pone.0136327.s008.docx]

**S8 File. Investigates whether Senate class is a moderator of the effect if positive and cited edits. In this case, we dichotomized Senate class into those senators who faced reelection in 2014 compared to all other senators.**

**Upcoming Elections as Moderator from Cox Regressions**

**Coefficient:**

**Positive**

*Coefficient:* 0.523*** 0.574***

*Standard error:* (0.089) (0.096)

**Positive X Upcoming Election**

*Coefficient:* 1.750* 1.557

*Standard error:*  (0.550) (0.485)

**Cited**

*Coefficient:* 0.577*** 0.507***

*Standard error:*  (0.112) (0.092)

**Cited X Upcoming Election**

*Coefficient:* 0.306*** 0.323***

*Standard error:*  (0.101) (0.104)

**Upcoming Election**

*Coefficient:*  0.794 2.504*** 0.783 2.146***

*Standard error:*  (0.178) (0.607) (0.203) (0.626)

**N**  300 200 300 200

**Fixed Effects** Yes Yes Yes Yes

**Covariates** No No Yes Yes

**Studies**  1,2,4 1,4 1,2,4 1,4

Note: *Significant at the 10% level; **Significant at the 5% level; ***Significant at the 1% level; Fixed Effects refer to fixed effects for study wave; Covariates Yes means controlling for the date and time order in which an edit was randomly assigned to be made, a binary variable for Republicans, Senate class, region (NE, S, W), length of incumbency, log of Wikipedia page character count before Study 1 began, log of state population, and a dichotomous influence variable for party leaders and committee chairs.
