# Supplementary material for: Editorial Bias in Crowd-Sourced Political Information
Source: PLoS One. 2015 Sep 2;10(9):e0136327. doi: 10.1371/journal.pone.0136327 (PMC4558055; doi:10.1371/journal.pone.0136327)
Supplement: S9 File — (DOCX) [file pone.0136327.s009.docx]

**S9 File. Examines whether the other covariates included in our registered pre-analysis plan similarly moderate the effect of either positive (Table A) or cited (Table B) edits.**

**Table A: Covariates as Moderators of Positive Edits from Cox Regressions**

**Coefficient:**

**Positive X Republican**

*Coefficient:* 1.115 1.160

*Standard error:*  (0.333) (0.300)

**Positive X Influential**

*Coefficient:* 0.905 0.906

*Standard error:*  (0.368) (0.312)

**Positive X NE Region**

*Coefficient:* 0.316*** 0.472*

*Standard error:*  (0.149) (0.192)

**Positive X S Region**

*Coefficient:* 0.893 0.973

*Standard error:*  (0.334) (0.314)

**Positive X W Region**

*Coefficient:* 1.019 1.075

*Standard error:*  (0.386) (0.364)

**Positive X Length of Incumbency**

*Coefficient:* 0.989 0.981

*Standard error:*  (0.019) (0.017)

**Positive X Log(Page Length)**

*Coefficient:* 0.911 0.851

*Standard error:*  (0.254) (0.196)

**Positive X Log(State Population)**

*Coefficient:* 1.050 1.236

*Standard error:*  (0.171) (0.175)

**Wald test (p-value)** 0.321 0.221

**N**  300 400

**Main Effects** Yes Yes

**Fixed Effects** Yes Yes

**Covariates** Yes No

**Studies** 1,2,4 1,2,4,5

Note: *Significant at the 10% level; **Significant at the 5% level; ***Significant at the 1% level; Fixed Effects refer to fixed effects for study wave; Covariates Yes means controlling for the date and time order in which an edit was randomly assigned to be made, a binary variable for Republicans, Senate class, region (NE, S, W), length of incumbency, log of Wikipedia page character count before Study 1 began, log of state population, and a dichotomous influence variable for party leaders and committee chairs.

**Table B: Covariates as Moderators of Cited Edits from Cox Regressions**

**Coefficient:**

**Cited X Republican**

*Coefficient:* 0.797 0.993

*Standard error:*  (0.235) (0.270)

**Cited X Influential**

*Coefficient:* 2.055* 1.922*

*Standard error:*  (0.794) (0.687)

**Cited X NE Region**

*Coefficient:* 0.928 1.704

*Standard error:*  (0.497) (0.766)

**Cited X S Region**

*Coefficient:* 1.468 1.721

*Standard error:*  (0.532) (0.588)

**Cited X W Region**

*Coefficient:* 1.128 1.193

*Standard error:*  (0.417) (0.420)

**Cited X Length of Incumbency**

*Coefficient:* 1.056*** 1.056***

*Standard error:*  (0.020) (0.019)

**Cited X Log(Page Length)**

*Coefficient:* 0.906 0.922

*Standard error:*  (0.277) (0.228)

**Cited X Log(State Population)**

*Coefficient:* 0.992 1.139

*Standard error:*  (0.167) (0.169)

**Wald test (p-value)** 0.225 0.095

**N**  200 250

**Main Effects** Yes Yes

**Fixed Effects** Yes Yes

**Covariates** No No

**Studies** 1,4 1,2,4,5 (1^st^ Half)

Note: *Significant at the 10% level; **Significant at the 5% level; ***Significant at the 1% level; Fixed Effects refer to fixed effects for study wave; Covariates Yes means controlling for the date and time order in which an edit was randomly assigned to be made, a binary variable for Republicans, Senate class, region (NE, S, W), length of incumbency, log of Wikipedia page character count before Study 1 began, log of state population, and a dichotomous influence variable for party leaders and committee chairs.
