# Supplementary material for: Editorial Bias in Crowd-Sourced Political Information
Source: PLoS One. 2015 Sep 2;10(9):e0136327. doi: 10.1371/journal.pone.0136327 (PMC4558055; doi:10.1371/journal.pone.0136327)
Supplement: S10 File — (DOCX) [file pone.0136327.s010.docx]

**S10 File. Examines which covariates are predictive of survival time.**

**Covariates Predicting Length of Survival from Cox Regressions**

**Coefficient:**

**Republican**

*Coefficient:* 1.176

*Standard error:* (0.151)

**Influence**

*Coefficient:* 0.885

*Standard error:* (0.138)

**Incumbency Length**

*Coefficient:* 1.004

*Standard error:* (0.008)

**Log(Page Length)**

*Coefficient:* 0.988

*Standard error:* (0.120)

**Log(State Population)**

*Coefficient:* 1.059

*Standard error:* (0.075)

**NE Region**

*Coefficient:* 0.659**

*Standard error:* (0.136)

**S Region**

*Coefficient:* 0.841

*Standard error:* (0.132)

**W Region**

*Coefficient:* 0.882

*Standard error:* (0.147)

**N**  400

**Fixed Effects** No

**Studies** 1,2,4,5

Note: *Significant at the 10% level; **Significant at the 5% level; ***Significant at the 1% level; Fixed Effects refer to fixed effects for study wave
