# Supplementary material for: Editorial Bias in Crowd-Sourced Political Information
Source: PLoS One. 2015 Sep 2;10(9):e0136327. doi: 10.1371/journal.pone.0136327 (PMC4558055; doi:10.1371/journal.pone.0136327)
Supplement: S11 File — (DOCX) [file pone.0136327.s011.docx]

**S11 File. Ordinary Least Squares Robustness Check.**

As a final robustness check, we examined the ordinary least squares estimates on survival at 6, 12, and 24 hours as well as 7 days. These results, presented in Tables A and B estimate the likelihood that an edit was removed by that point in time. The OLS estimates consistently find that for active senators, positive and cited facts are less likely to have been removed while for retired and dead senators (Study 3) there is no difference.

**Table A: OLS Estimates on Survival at 6 Hours and 12 Hours**

**Coefficient: 6 hrs 6 hrs 6 hrs 12 hrs 12 hrs 12 hrs**

**Positive**

*Coefficient:* 0.113** -0.027 0.153*** -0.027

*Standard error:*  (0.049) (0.026) (0.052) (0.026)

**Cited**

*Coefficient:* 0.320*** 0.370***

*Standard error:*  (0.645) (0.065)

**N**  300 200 151 300 200 151

**Fixed Effects** Yes Yes No Yes Yes No

**Covariates** No No No No No No

**Studies** 1,2,4 1,4 3 1,2,4 1,4 3

Note: *Significant at the 10% level; **Significant at the 5% level; ***Significant at the 1% level; Fixed Effects refer to fixed effects for study wave; Covariates Yes means controlling for the date and time order in which an edit was randomly assigned to be made, a binary variable for Republicans, Senate class, region (NE, S, W), length of incumbency, log of Wikipedia page character count before Study 1 began, log of state population, and a dichotomous influence variable for party leaders and committee chairs.

**Table B: OLS Estimates on Survival at 24 Hours and 7 Days**

**Coefficient: 24 hrs 24 hrs 24 hrs 7 Days 7 Days 7 Days**

**Positive**

*Coefficient:* 0.147*** -0.027 0.127*** -0.054

*Standard error:* (0.052) (0.026) (0.047) (0.041)

**Cited**

*Coefficient:* 0.390*** 0.230***

*Standard error:*  (0.062) (0.053)

**N**  300 200 151 300 200 151

**Fixed Effects** Yes Yes No Yes Yes No

**Covariates** No No No No No No

**Studies** 1,2,4 1,4 3 1,2,4 1,4 3

Note: *Significant at the 10% level; **Significant at the 5% level; ***Significant at the 1% level; Fixed Effects refer to fixed effects for study wave; Covariates Yes means controlling for the date and time order in which an edit was randomly assigned to be made, a binary variable for Republicans, Senate class, region (NE, S, W), length of incumbency, log of Wikipedia page character count before Study 1 began, log of state population, and a dichotomous influence variable for party leaders and committee chairs.
