# Supplementary material for: Editorial Bias in Crowd-Sourced Political Information
Source: PLoS One. 2015 Sep 2;10(9):e0136327. doi: 10.1371/journal.pone.0136327 (PMC4558055; doi:10.1371/journal.pone.0136327)
Supplement: S12 File — (DOCX) [file pone.0136327.s012.docx]

**S12 File. Mechanical Turk Analysis of Facts**

After we completed all five studies, Amazon Mechanical Turk was used to evaluate how the facts were perceived. Each fact was rated five separate times on how positive or negative it is, how relevant it is, and how favorable the survey respondent felt towards the senator after reading the fact. Consistent with our expectations, we find that the positive facts collected by the undergraduate research assistants were perceived by survey respondents on Mechanical Turk as being more positive, equally relevant, and leading to more favorable feelings towards the senator than the negative facts.

*Description of Survey*

Workers on Mechanical Turk were given the following set of instructions: “Please carefully read the consent statement and the following political fact. After you read it, we will ask you to evaluate it for: How positive, negative, or neutral you think the fact is, How relevant the fact is for your understanding of this politician, and How this fact makes you feel about the politician. This fact is an incomplete snapshot of a politician. Please answer honestly and based only on your impression of this fact. There is no right or wrong answer.” After reading a consent statement, the Mechanical Turk workers were presented with the fact and the following three questions:

- Would you consider this fact to be positive, negative, or neither positive nor negative?
  - Very Positive
  - Positive
  - Somewhat Positive
  - Neither Positive nor Negative
  - Somewhat Negative
  - Negative
  - Very Negative
- Is this fact relevant, irrelevant or neither relevant nor irrelevant to your understanding of the politician?
  - Very Relevant
  - Relevant
  - Somewhat Relevant
  - Neither Relevant nor Irrelevant
  - Somewhat Irrelevant
  - Irrelevant
  - Very Irrelevant
- After reading this fact, do you have a favorable, unfavorable, or neither favorable nor unfavorable view of this politician?
  - Very Favorable
  - Favorable
  - Somewhat Favorable
  - Neither Favorable nor Unfavorable
  - Somewhat Unfavorable
  - Unfavorable
  - Very Unfavorable

Mechanical Turk workers were paid $0.10 per fact coded and were given an opportunity to code up to 50 facts. For each worker, facts were randomly sampled without replacement from the set of 551 facts included in the five studies, plus an additional 49 facts that were seeded as quality control (e.g., Julie Green was an award-winning politician. If you are reading this fact, please select the second, last, and then second options for the three questions.).

*Results*

First, on the quality control facts, they were coded 245 times (49 facts, 5 times each). Of these, 237 (96.7%) were coded according to the directions, suggesting that the Mechanical Turk workers were paying attention and carefully reading the facts.

When we calculate the inter-rater reliability across the facts and five raters, we find fairly strong agreement. On the positive-negative question, the Fleiss’s kappa across all seven categories is 0.20 and 0.56 when collapsed to three categories. On the relevance question, the Fleiss’s kappa across all seven categories is 0.04 and 0.08 when collapsed to three categories. On the favorability question, the Fleiss’s kappa across all seven categories is 0.13 and 0.38 when collapsed to three categories.

When we compare the research assistants’ coding of the facts to the coding of the Mechanical Turk workers, we find that the facts collected to be positive by the undergraduate research assistants were perceived by the Mechanical Turk Workers as more positive, equally relevant, and leading to more favorable feelings towards the senator than the negative facts. We see this in the following three box plots, Figures A, B, and C.

*Figure A. Distribution of Mechanical Turk responses on how positive or negative the facts used were perceived to be.*

*Figure B. Distribution of Mechanical Turk responses on how relevant or irrelevant the facts used were perceived to be.*

*Figure C. Distribution of Mechanical Turk responses on how favorable or unfavorably the senator seemed after reading the fact.*

As further robustness checks, Table A presents regression results of the Mechanical Turk 7-point coding (higher=more positive, more relevant, and more favorable) regressed on the binary variable as to whether the fact was used as either a positive or negative fact in the five studies. Consistent with the box-plots, we see that positive facts were perceived to be more positive, equally relevant, and more favorable at statistically significant levels. As an additional robustness check designed to guard against any desirability bias and learning that may occur as Mechanical Turk workers rate up to 50 of these facts, we produce the same regression using only the first randomly-assigned fact that each worker saw. The results are substantively the same.

*Table A. Regression Analysis of Mechanical Turk Coding*

|  | Positive-Negative | | Relevance | | Favorability | |
| --- | --- | --- | --- | --- | --- | --- |
|  | All | First Fact | All | First Fact | All | First Fact |
| Positive | 2.96  (0.08) | 3.26  (0.23) | -0.03  (0.07) | 0.03  (0.25) | 2.30  (0.09) | 2.08  (0.24) |
| *N* | 2751 | 112 | 2754 | 113 | 2753 | 113 |

*Note:* For each question, the first column reports the regression across all facts and the second column is only for the first fact seen. The first number reported is the bivariate regression coefficient and the second number, in parentheses, is the standard error clustered at the level of the worker (since each worker could rate up to 50 facts). The *N*’s vary slightly due to missing responses.
